# Cancer-associated fibroblasts-mediated ATF4 expression promotes malignancy and gemcitabine resistance in pancreatic cancer via the TGF-β1/SMAD2/3 pathway and ABCC1 transactivation

**DOI:** 10.1038/s41419-021-03574-2

**Published:** 2021-03-29

**Authors:** Lusheng Wei, Qing Lin, Yanan Lu, Guolin Li, Leyi Huang, Zhiqiang Fu, Rufu Chen, Quanbo Zhou

**Affiliations:** 1 Guangdong Provincial Key Laboratory of Malignant Tumor Epigenetics and Gene Regulation, Sun Yat-sen Memorial Hospital, Sun Yat-sen University, Guangzhou, Guangdong 510120 China; 2 Department of Pancreatobiliary Surgery, Sun Yat-sen Memorial Hospital, Sun Yat-sen University, Guangzhou, Guangdong 510120 China; 3 Department of General Surgery, Guangdong Provincial People’s Hospital, Guangdong Academy of Medical Sciences, Guangzhou, Guangdong 510055 China; 4 Department of Anesthesiology, Sun Yat-sen Memorial Hospital, Sun Yat-sen University, Guangzhou, Guangdong 510120 China

**Keywords:** Cancer microenvironment, Cancer therapeutic resistance, Pancreatic cancer

## Abstract

Cancer-associated fibroblasts (CAFs) contribute to malignant progression and chemoresistance in pancreatic ductal adenocarcinoma (PDAC). However, little is known about the underlying mechanism. In this study, we investigated the potential role and mechanisms of activating transcription factor 4 (ATF4) in CAFs-induced malignancy and gemcitabine resistance. We demonstrated that ATF4 is overexpressed in PDAC and associated with a poor prognosis. Silencing ATF4 expression decreased proliferation, colony formation, migration, gemcitabine sensitivity, and sphere formation. Subsequently, we revealed that CAFs secrete TGF-β1 to upregulate the expression of ATF4 in PDAC cells via the SMAD2/3 pathway and induce cancer progression, cancer stemness, and gemcitabine resistance. Furthermore, we demonstrated that ATF4 directly binds to the ABCC1 promoter region to activate transcription. In summary, these data demonstrate that CAFs contribute to malignancy and gemcitabine resistance in PDAC by upregulating the expression of ATF4 via the TGF-β1/SMAD2/3 axis and highlight that ATF4 is an attractive therapeutic target for combating gemcitabine resistance in PDAC.

## Introduction

Pancreatic ductal adenocarcinoma (PDAC) is the fourth leading cause of cancer-related death in the United States, with a dismal 5-year survival rate of ∼10% (ref. ^1^). Because of the absence of early symptoms, most patients with PDAC are diagnosed in the advanced unresectable stage. Only 15–20% of cases are diagnosed in the early resectable stages, and the 5-year survival rate of patients who undergo surgical resection is only ~20% (ref. ^2,3^). The effect of gemcitabine-based chemotherapy is limited for all stages of PDAC (ref. ^4,5^). Currently, the tumor microenvironment is suggested to play an important role in the highly malignant biological behavior of pancreatic cancer^6^. Therefore, it is urgent to delve into the mechanisms of intricate cellular communication to develop effective therapeutic strategies.

Cancer-associated fibroblasts (CAFs) are one of the most abundant cells in the tumor microenvironment of pancreatic cancer. CAFs, which express activated myofibroblast markers including α-smooth muscle actin (α-SMA), fibroblast activation protein (FAP), and fibroblast-specific protein 1, stem from pancreatic stellate cells, quiescent resident fibroblasts, and mesenchymal stem cells. CAFs participate in the regulation of tumorigenesis, progression, metastasis, and drug resistance in various cancers, including pancreatic cancer, by secreting exosomes and cellular factors^7,8^. Activating transcription factor 4 (ATF4), a basic region-leucine zipper transcription factor functions as a master regulator of stress responses and a developmental regulator by binding to C/EBP-ATF response elements in the promoter regions of target genes and activating transcription^9,10^. ATF4 is overexpressed in tumors, including breast cancer^11^, neuroblastoma^12^, and osteoblasts^13^, and participates in regulating tumor growth, autophagy, drug resistance, and metastasis^14–17^. ATF4 has been reported to participate in regulating other genes in pancreatic cancer^17,18^. However, the specific roles of ATF4 in CAFs promoting pancreatic cancer progression and the mechanism of gemcitabine resistance are still poorly elucidated.

Here, we revealed that CAFs secrete TGF-β1 to upregulate the expression of ATF4 in PDAC cells via the SMAD2/3 pathway. ATF4 is associated with poor prognosis in PDAC patients, and silencing ATF4 inhibits proliferation, migration, cancer stemness, and gemcitabine resistance. Mechanistically, we demonstrated that ATF4 directly binds to the promoter region of ABCC1 and activates the transcript, resulting in gemcitabine resistance. These data suggest that targeting ATF4 may provide a novel therapeutic option for reversing clinical PDAC gemcitabine resistance.

## Results

### ATF4 is overexpressed in pancreatic cancer and is associated with a poor prognosis

**Fig. 1:**
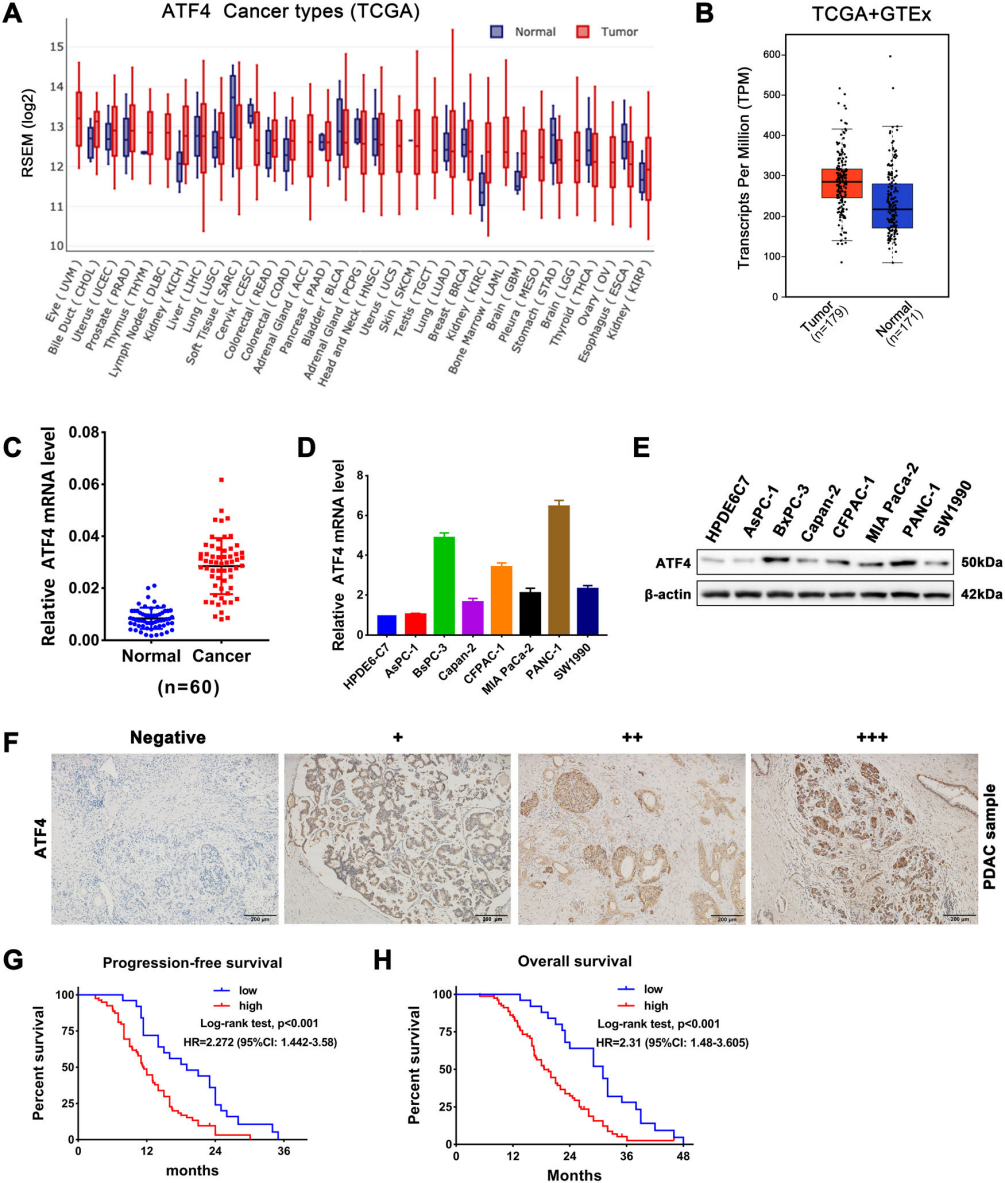
ATF4 is overexpressed in pancreatic cancer and is associated with poor prognosis in patients with PDAC. **A** ATF4 expression profile across all tumor samples and corresponding normal tissues in the TCGA database. **B** Combined analysis of ATF4 expression in pancreatic cancer tissues and corresponding normal tissues from TCGA and GTEx databases. **C** qRT-PCR analysis of ATF4 expression levels in 60 PDAC tissue samples and paired normal adjacent tissue samples collected in our hospital. ^***^p < 0.001. **D** qRT-PCR assay was performed to detect the mRNA expression of ATF4 in the pancreatic normal ductal epithelial cell line HPDE6-C7 and cancer lines including AsPC-1, BxPC-3, Capan-2, CFPAC-1, MIA PaCa-2, PANC-1, and SW1990. *n* = 3. **E** Western blot assay showing the protein expression level of ATF4 in a pancreatic normal ductal epithelial cell line and cancer cell lines. **F** Representative IHC staining images showed negative (−), weakly positive (+), moderate positive (++), and strongly positive (+++) expression of ATF4 in pancreatic cancer tissue samples. **G** Kaplan–Meier survival analysis was performed to compare ATF4-high and ATF4-low PDAC patients’ progression-free survival times from our hospital. *N* = 52, log-rank test. **H** Kaplan–Meier survival analysis was performed to compare ATF4-high and ATF4-low PDAC patients’ overall survival times from our hospital. *N* = 52, log-rank test.

To investigate the role of ATF4 in pancreatic cancer progression, we first analyzed ATF4 expression in pancreatic cancer tissues and cancer cell lines and analyzed its correlation with pancreatic cancer patient prognosis. The results of the cancer genome atlas (TCGA) database^19^ analysis showed that ATF4 expression was upregulated in 21 types of tumors compared with that in the corresponding normal tissues among 31 types of tumors (Fig. 1A). Combined analysis of data from TCGA and the genotype-tissue expression (GTEx) database showed that the expression of ATF4 was significantly upregulated in pancreatic cancer tissues (Fig. 1B). In addition, we performed qRT-PCR assays on 60 PDAC tissue samples and matched normal adjacent tissue samples, and the results showed that ATF4 was significantly overexpressed in pancreatic cancer tissues (Fig. 1C). Compared with the normal pancreatic ductal epithelial cell line HPDE6-C7, ATF4 was significantly upregulated in pancreatic cancer cell lines such as PANC-1, BxPC-3, CFPAC-1, MIA PaCa-2, and SW1990 (Fig. 1D, E). The results of the IHC assay also showed that ATF4 staining was positive in pancreatic cancer tissue samples (Figs. S1 and 1F). Moreover, statistical analysis demonstrated that the ATF4 expression level in PDAC patients was correlated with differentiation, tumor stage, and neural invasion (Table 1). Univariate and multivariate analyses revealed that ATF4 expression was an independent prognostic factor (Table 2). Furthermore, Kaplan–Meier survival analysis showed that lower ATF4 expression levels were correlated with longer progression-free survival times (PFS) and overall survival times (OS) in patients with PDAC (Fig. 1G, H). These results indicated that ATF4 was overexpressed in pancreatic cancer and was associated with poor prognosis in PDAC patients.

**Table 1:** The correlation between ATF4 expression level and the clinicopathological features of matched PDAC patients.

Pathological feature	ATF4 expression level		χ^2 a^	*p* value
	Low (*n* = 25)	High (*n* = 79)		
Sex			0.004	0.949
Male	16	50		
Female	9	29		
Age			0.081	0.776
<60	9	26		
≥60	16	53		
Differentiation			6.063	0.048*
Well	12	18		
Moderate	9	48		
Poor	4	13		
AJCC stage^b^			0.576	0.750
I	5	11		
II	16	53		
III	4	15		
Tumor stage			7.993	0.046*
T1	10	11		
T2	10	37		
T3	3	18		
T4	2	13		
Lymph node stage			3.059	0.217
N0	14	30		
N1	7	37		
N2	4	12		
Neural invasion			6.964	0.008**
Yes	12	60		
No	13	19		

^a^Chi-square test.

^b^American Joint Committee on Cancer (AJCC), patients were staged in accordance with the eighth edition of AJCC cancer’s TNM classification. *p < 0.05, **p < 0.01.

**Table 2:** Univariate and multivariate analysis of overall survival in PDAC patients (*n* = 104).

Variable	Feature	Univariate analysis (log-rank)			Multivariate analysis (Cox)		
		HR	95% CI	*P* value	HR	95% CI	*P* value
Sex	Male	1.034	0.651–1.641	0.888			
	Female						
Age	<60	1.032	0.814–1.308	0.795			
	≥60						
Differentiation	Well			0.784			
	Moderate	1.116	0.819–1.521	0.486			
	Poor	0.911	0.601–1.381	0.660			
AJCC stage	I			0.881			
	II	0.982	0.625–1.543	0.937			
	III	0.932	0.671–1.295	0.676			
Tumor stage	T1			0.412*			0.314
	T2	1.002	0.651–1.542	0.993	1.352	0.830–2.204	0.226
	T3	1.339	0.951–1.886	0.094	1.257	0.886–1.784	0.199
	T4	0.891	0.567–1.400	0.617	0.876	0.554–1.386	0.571
Lymph node stage	N0			0.234*			0.530
	N1	0.97	0.702–1.340	0.854	1.120	0.801–1.566	0.509
	N2	1.325	0.959–1.832	0.88	1.145	0.817–1.606	0.432
Neural invasion	yes	0.812	0.635–1.039	0.098*	0.975	0.748–1.272	0.855
	no						
ATF4 expression	low	1.515	1.144–2.006	0.004**	1.625	1.155–2.286	0.005**
	high						

*HR* hazard ratio, *95% CI* confidence interval, *Cox regression analysis* Method: Enter.

*p < 0.05, **p < 0.01.

### Silencing ATF4 inhibits the proliferation, migration, and invasion of pancreatic cancer cells

**Fig. 2:**
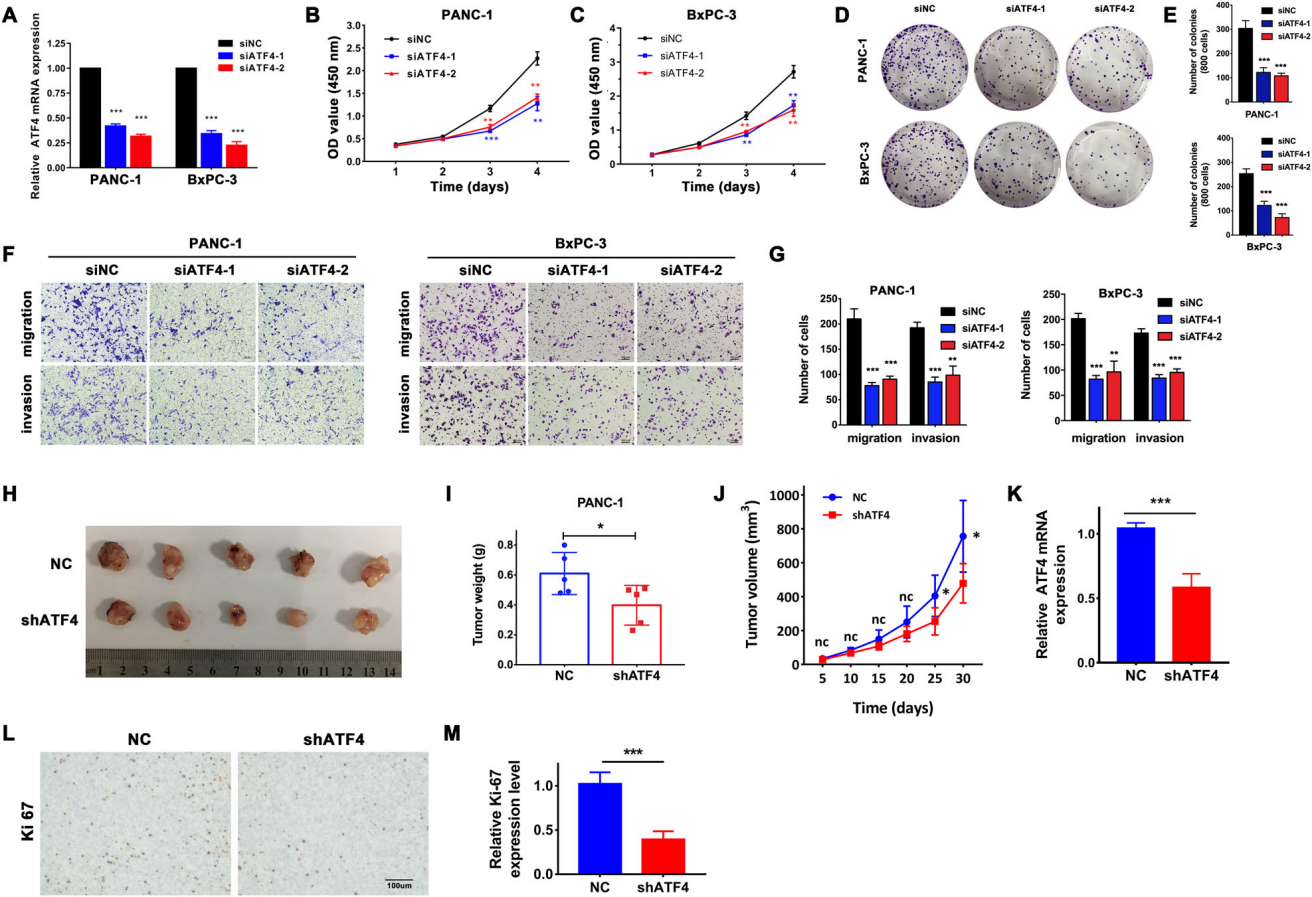
Silencing ATF4 expression inhibits the proliferation, migration, and invasion of pancreatic cancer cells in vitro and inhibits tumor growth in vivo. **A** qRT-PCR assay was performed to detect the silencing effect of ATF4 siRNA in PANC-1 and BxPC-3 cells. *n* = 3, ^***^p < 0.001. **B**, **C** CCK8 analysis showed the growth curve of PANC-1 and BxPC-3 cells transfected with ATF siRNA. *n* = 3, ^**^p < 0.01, ^***^p < 0.001. **D**, **E** Plate colony formation assays showed that colony formation decreased in PANC-1 and BxPC-3 cells with ATF4 silencing. *n* = 3, ^**^p < 0.01, ^***^p < 0.001. **F**, **G**. Transwell assays showed the fractions of migrated and invaded PANC-1 and BxPC-3 cells. Silencing ATF4 expression decreased the migration of invaded pancreatic cancer cells. *n* = 3, ^**^p < 0.01, ^***^p < 0.001. **H** Image of harvested tumors from PANC-1 cells with stable ATF4 knockdown or mock cells subcutaneously transplanted into the bilateral hind legs of nude mice. **I** The tumor weights are shown as the mean ± SD, *n* = 5. ^*^p < 0.05. **J** The tumor growth curve. The points and bars represent the mean ± SD. *n* = 5, ns not significantly different. ^*^p < 0.05. **K** qRT-PCR assay analyzed the mRNA expression level of ATF4 in tumor tissues from shATF4 PANC-1 cells compared with mock cells. *n* = 5, ^***^p < 0.001. **L**, **M** IHC staining showed that the positive expression rate of Ki67 was lower in tumor samples from shATF4 PANC-1 cells than in mock cells. *n* = 5, ^***^p < 0.001.

To investigate the role of ATF4 in pancreatic cancer cell proliferation and migration, CCK8, plate colony formation, Transwell migration, and Transwell invasion assays were performed. First, PANC-1 and BxPC-3 cells were transfected with siRNA (Fig. 2A). The CCK8 assay showed that silencing ATF4 suppressed pancreatic cancer PANC-1 and BxPC-3 cell growth (Fig. 2B, C). Meanwhile, plate colony formation assays showed that downregulation of ATF4 inhibited colony formation in PANC-1 and BxPC-3 cells (Fig. 2D, E). Then, Transwell assays were performed to detect the role of ATF4 expression in regulating the migration and invasion of pancreatic cancer cells. The results showed that silencing ATF4 expression downregulated the migration ability of PANC-1 and BxPC-3 pancreatic cancer cells (Fig. 2F, G). These experiments indicated that ATF4 participated in regulating the cellular proliferation and migration capability of pancreatic cancer.

### Knockdown of ATF4 inhibits pancreatic cancer growth in vivo

To further investigate whether ATF4 played a role in tumor growth in vivo, we performed a subcutaneous injection of PANC-1 cells with stable ATF4 knockdown (PANC-1 shATF4) or mock-transfected cells (shNC) into the bilateral hind legs of athymic nude mice. A month later, xenografted tumors all developed at the injection site of all mice. Consistent with the in vitro results, xenografted tumors grown in PANC-1 shATF4 cells had a smaller mean size and weight than xenografted tumors grown in mock cells (Fig. 2H–J). The qRT-PCR analysis confirmed ATF4 knockdown in xenotransplanted tumors in the shATF4 group (Fig. 2K). Then, IHC staining showed a positive expression of Ki67, an objective index to evaluate the cell proliferation state, which was lower in the shATF4 group than in the shNC group (Fig. 2L, M). These findings suggested that ATF4 participated in regulating the proliferation of pancreatic cancer cells in mouse xenograft models.

### Silencing ATF4 increases sensitivity to gemcitabine and suppresses the stemness of pancreatic cancer cells

**Fig. 3:**
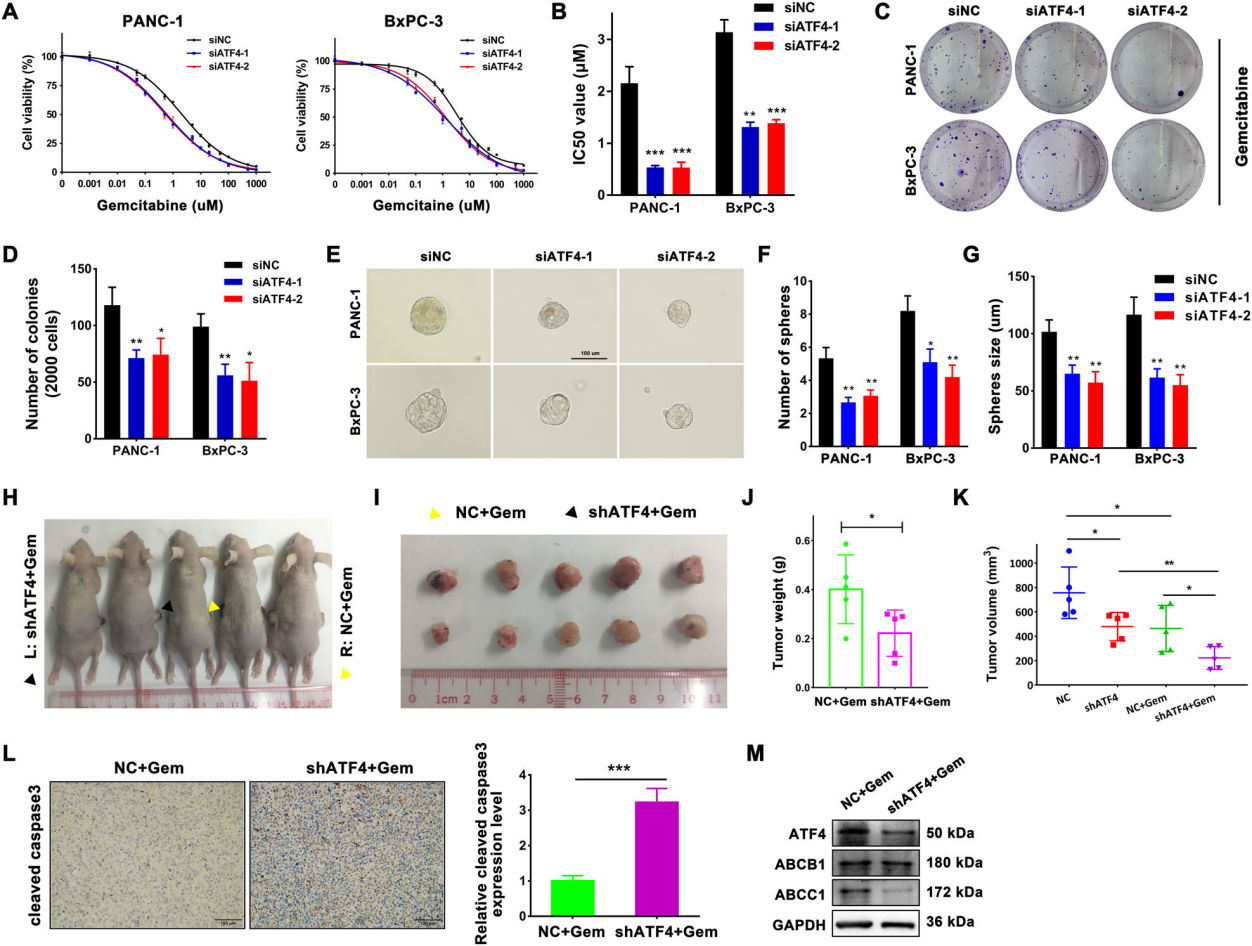
Silencing of ATF4 increases sensitivity to gemcitabine and inhibits the stemness of pancreatic cancer cells. **A** CCK8 assay showed the survival rate of PANC-1 and BxPC-3 cells treated with various concentrations of gemcitabine. The fitted curve was analyzed with GraphPad Prism 7 software according to the values of survival rate. *n* = 5. **B** IC50 values were calculated with GraphPad Prism 7 software. *n* = 3. ^**^p < 0.01, ^***^p < 0.001. **C** The cell colony formation assays were performed to detect the capability of colony formation with gemcitabine treatment at the concentration of 50 nM. **D** The bar chart shows the number of colonies of PANC-1 and BxPC-3 cells with or without ATF4 silencing. **E** Sphere forming assays were performed to analyze the stem cell properties of PANC-1 and BxPC-3 cells with or without ATF4 silencing. **F**, **G** The bar chart shows that silencing ATF4 decreased the number of formed spheres and size of formatted spheres. *n* = 3. ^**^p < 0.01. **H**, **I**. PANC-1 cells with stable ATF4 knockdown or mock cells were subcutaneously transplanted into the bilateral hind legs of nude mice. One week after transplantation, all mice received an intraperitoneal injection of gemcitabine at a concentration of 100 mg/kg twice a week. PANC-1 cells with stable knockdown of ATF4 (black triangle) or mock cells (yellow triangle) developed primary tumors. **H** Image of harvested tumors. **I** The tumor growth curve. The points and bars represent mean ± SD. *n* = 5, ^*^p < 0.05. **J** The tumor weights are shown as the mean ± SD. *n* = 5, ^*^p < 0.05. **K** Volume of xenograft tumors with or without gemcitabine treatment. **L** IHC staining showed increased positive expression of cleaved caspase-3 in tumor tissues from shATF4 PANC-1 cells. *n* = 5, ^***^p < 0.001. **M** Western blot assay detected the protein expression of ATF4 and ABCC1 in tumor samples from shATF4 PANC-1 cells or mock cells.

Gemcitabine has been the fundamental chemotherapy drug for PDAC patients in all stages of disease for the last two decades. Resistance to anticancer drugs severely limits the effectiveness of chemotherapy. To determine whether ATF4 expression correlates with gemcitabine resistance, we detected the half-maximal inhibitory concentration (IC50) of gemcitabine in PANC-1 and BxPC-3 cells. Downregulation of ATF4 levels decreased the IC50 value of gemcitabine, suggesting that silencing ATF4 expression can enhance sensitivity to gemcitabine in pancreatic cancer cells (Fig. 3A, B). In the meantime, the cell colony formation assay showed that downregulating ATF4 decreased the number of colonies of PANC-1 and BxPC-3 cells with gemcitabine treatment at the concentration of 50 nM (Fig. 3C, D). The existence of cancer stem cells is a vital mechanism for chemotherapy resistance. A sphere formation assay was performed to analyze the capability of pancreatic cancer cells to transform into cancer stem cells. After ATF4 expression was downregulated by siRNA, the sphere number and size were suppressed in PANC-1 and BxPC-3 cells (Fig. 3E–G). These results indicated that silencing ATF increases the sensitivity to gemcitabine in pancreatic cancer cells.

### Knockdown of ATF4 enhances the anticancer effectiveness of gemcitabine for pancreatic cancer in vivo

To confirm the role of ATF4 in the anticancer effectiveness of gemcitabine in pancreatic xenografted tumor models in vivo, we performed an intraperitoneal injection of gemcitabine at a concentration of 100 mg/kg twice a week one week after subcutaneous xenotransplanted tumor models were established successfully with PANC-1 shATF4 or PANC-1 shNC cells. One month later, all mice were sacrificed, and the tumors were preserved for further experiments (Fig. 3H). The xenografted tumors had a smaller size and a small weight in the shATF4 group than in the shNC group (Fig. 3I, J) under gemcitabine treatment. Then, we analyzed cleaved caspase-3 expression, an objective index to evaluate the cell apoptosis state, in xenografted tumors. The results of IHC staining showed that cleaved caspase-3 expression was significantly higher in the shATF4 group than in the shNC group (Fig. 3L). Furthermore, the western blot assay showed that protein expression levels of ATF4, ABCC1 were downregulated in the shATF4 group under gemcitabine treatment (Fig. 3M). Figure 3K showed the volumes of xenograft tumors with or without gemcitabine treatment. According to the Zhengjun Jin Method to evaluate the combination efficacy^20^, the *Q* value of combining ATF4 silencing and gemcitabine treatment was 1.17 (>1.15), meaning the synergistic effect between silencing ATF4 and gemcitabine treatment. These results indicated that downregulation of ATF4 enhances chemosensitivity to gemcitabine in pancreatic cancer in vivo.

### CAFs increase the malignancy and gemcitabine resistance of pancreatic cancer cells by upregulating ATF4 expression

**Fig. 4:**
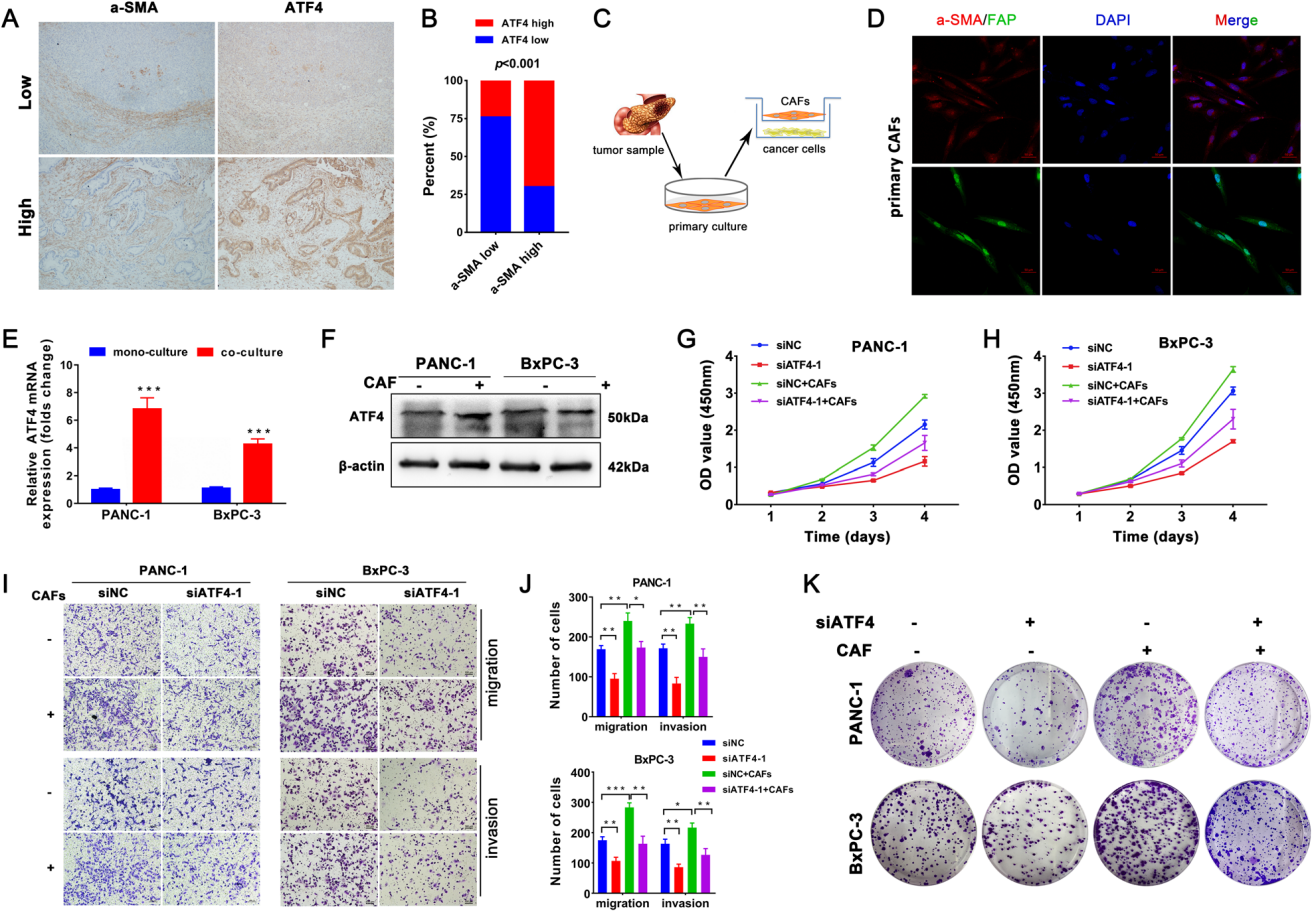
CAFs promote the process of pancreatic cancer cells via upregulation of ATF4 expression. **A** IHC staining of serial sections analyzed the expression of a-SMA and ATF4. **B** The chart shows the correlation between a-SMA expression and ATF4 expression. **C** The schematic diagram of the CAF coculture system with pancreatic cancer cells. **D** Immunofluorescence staining showed a-SMA and FAP were expressed in isolated CAFs. **E**, **F** qRT-PCR and western blot assays were used to analyze the mRNA and protein levels of ATF4 in PANC-1 and BxPC-3 cells cocultured with CAFs. *n* = 3. ^**^p < 0.01, ^***^p < 0.001. **G**, **H**. A CCK8 assay was performed to analyze the cellular growth curve in PANC-1 and BxPC-3 cells cocultured with CAFs, with or without siATF4. **I**, **J** Transwell assays analyzed the migration and invasion of PANC-1 and BxPC-3 cells cocultured with CAFs with or without siATF4. *n* = 3. ^*^p < 0.05, ^**^p < 0.01, ^***^p < 0.001. **K** Cell colony formation assays analyzed the colony formation capability of PANC-1 and BxPC-3 cells cocultured with CAFs with or without siATF4.

Pancreatic cancer is characterized by a highly fibrotic desmoplastic stroma. CAFs are the most abundant cells in the tumor microenvironment of pancreatic cancer and participate in facilitating tumor progression and impairing intratumoral drug delivery^21^. To investigate the potential relationship between the infiltration of CAFs and the expression of ATF4, we performed IHC staining for α-SMA, a marker of CAFs, and ATF4 with serial sections from the same PDAC sample. IHC results of serial sections showed that 70% of PDAC patients who highly expressed α-SMA also highly expressed ATF4 (Fig. 4A, B). Then, primary normal fibroblasts (NFs) and CAFs were isolated from fresh normal pancreatic tissues and pancreatic tumor samples of PDAC patients, respectively (Fig. 4C), as reported in our previous study^22^. Compared with NFs, the isolated CAFs highly expressed α-SMA and FAP, as confirmed by immunofluorescence staining (Figs. 4D and S2). qRT-PCR and western blot assays showed that the protein and mRNA expression levels of ATF4 were upregulated in PANC-1 and BxPC-3 cells after coculture with CAFs (Fig. 4E, F). CCK8 analysis showed that CAFs promoted the growth of pancreatic cancer cells, but silencing ATF4 partially inhibited the growth-promoting effect of CAFs (Fig. 4G, H). After coculture with CAFs, the number of migrated and invaded PANC-1 and BxPC-3 cells increased, and CAF-induced effects were partially inhibited by silencing ATF4 (Fig. 4I, J). Coculture with CAFs and silencing of ATF4 in PANC-1 and BxPC-3 cells showed similar effects in cell colony formation assays (Fig. 4K). Taken together, these analyses suggested that CAFs accelerate the malignant progression of pancreatic cancer via the upregulation of ATF4.

### TGF-β1 secreted from CAFs mediates the upregulation of ATF4 in pancreatic cancer cells via the SMAD2/3 pathway

**Fig. 5:**
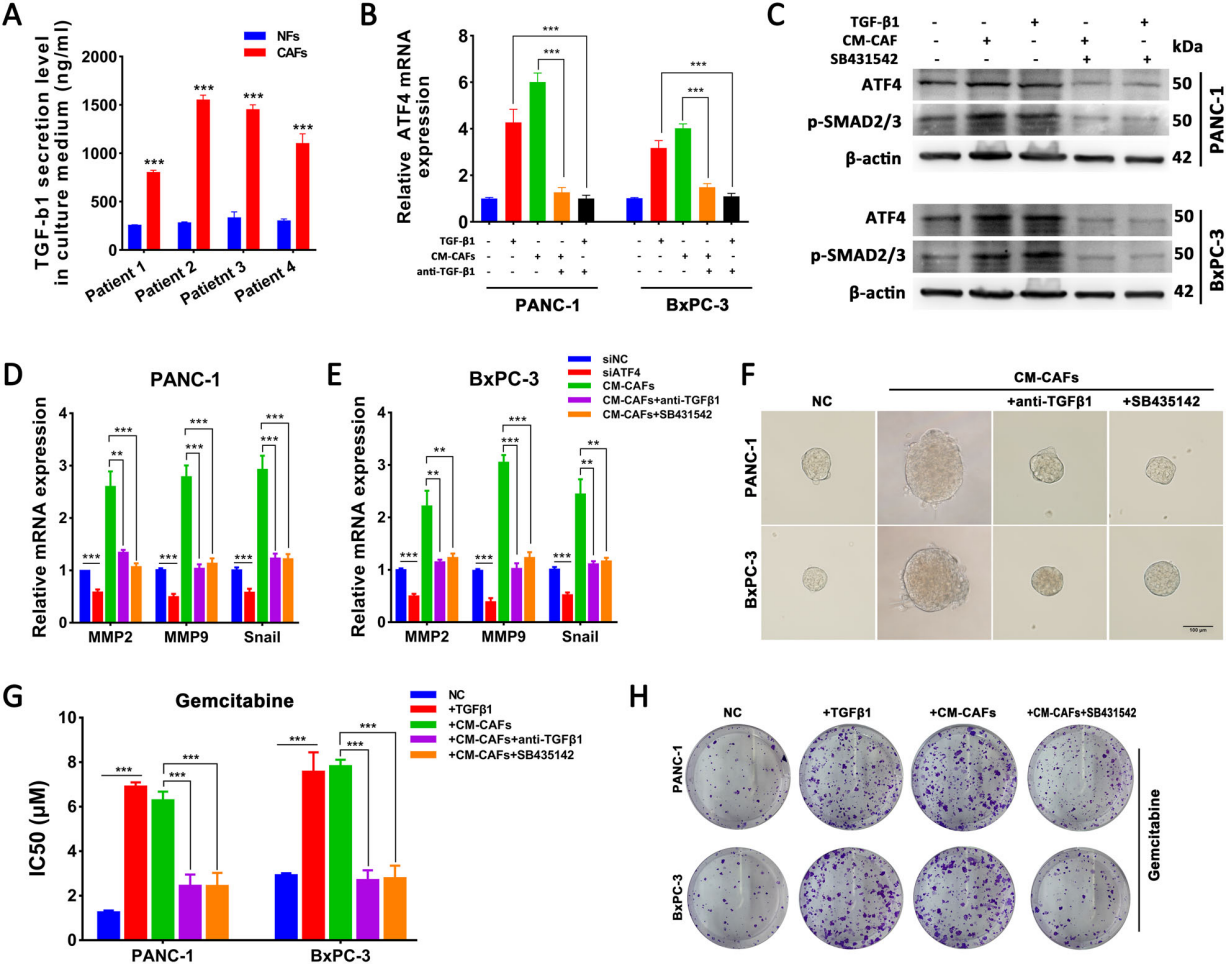
TGF-β1 secreted from CAFs upregulates ATF4 expression via the SMAD2/3 pathway. **A** ELISA showed higher secretion of TGF-β1 in CM-CAFs than in NFs. **B** qRT-PCR assays showed that TGF-β1 and CM-CAFs treatment upregulated the ATF4 expression level, but neutralization with anti-TGF-β1 reversed this effect. **C** Western blot assay showed that TGF-β1 and CM-CAFs treatment increased the level of ATF4 and p-SMAD2/3, but SB431542, an ALK5 inhibitor, reversed this effect. **D**, **E** qRT-PCR assays were performed to analyze the expression of MMP2, MMP9, and Snail under different treatment conditions. **F** Sphere formation assay showed that CM-CAFs treatment enhanced cancer stem cell sphere formation but suppressed when anti-TGF-β1 or SB431542 was added to CM-CAFs. **G** IC50 values in PANC-1 and BxPC-3 cells were analyzed with GraphPad Prism 7 after performing a CCK8 assay under various concentrations of gemcitabine. ^***^p < 0.001. **H** Cell colony formation assays analyzed the gemcitabine resistance of PANC-1 and BxPC-3.

Since CAFs enhance tumorigenesis, metastasis, or drug resistance in various cancer cells via secretion of soluble factors^23^, we detected the mRNA expression of FGF2, TGF-β1, SDF-1, IL-6, IGF-1, CCL18, PDGF-AA, HGF, and TNF-α and confirmed that CAFs highly expressed these cellular factors, especially TGF-β1 and SDF-1 (Fig. S3A). Maupin et al. reported genomic changes in the effect of TGF-β1 on PANC-1 cells^24^, and the dataset revealed that ATF4 was upregulated in the TGF-β1-treated group (Fig. S3B). ELISA analysis showed that CM from CAFs contained higher levels of TGF-β1 than CM from NFs (Fig. 5A). After TGF-β1 or CM-CAFs treatment, PANC-1 and BxPC-3 cells significantly upregulated ATF4 mRNA and protein expression levels, and the promoting effect of CAFs were inhibited by the neutralizing antibody anti-TGF-β1 (Fig. 5B, C). To confirm the role of the TGF-β1 pathway in regulating ATF4 expression, SB431542, a specific ALK5 inhibitor, was added to PDAC cells treated with TGF-β1 or CM-CAFs. Treatment with TGF-β1 and CM-CAFs increased the level of p-SMAD2/3, and SB431542 inhibited the phosphorylation of SMAD2/3 (Fig. 5C). The qRT-PCR analysis showed that CM-CAFs treatment upregulated the expression of MMP2, MMP9, and Snail, but anti-TGF-β1 and SB431542 inhibited the regulatory effect of CM-CAFs (Fig. 5D, E). Furthermore, sphere formation analysis, gemcitabine IC50 analysis, and plate colony formation analysis also revealed that anti-TGF-β1 and SB431542 reversed the promoting effect of CM-CAFs (Fig. 5F–H). Taken together, these findings indicate that CAFs-secreted TGF-β1 activates the SMAD2/3 pathway to upregulate the expression of ATF4, which is attributed to the regulation of malignancy and gemcitabine sensitivity in PDAC cells.

### ATF4 transactivates and directly binds to the ABCC1 promoter

**Fig. 6:**
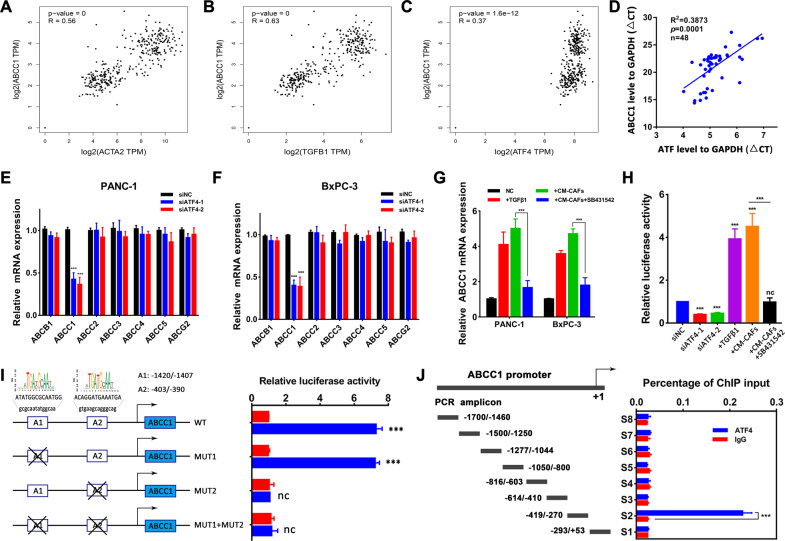
ATF4 directly binds to the ABCC1 promoter region to activate transcription. **A**–**C** Correlation analysis between ACTA2, TGFB1, ATF4 expression, and ABCC1 expression using the gene expression profiling interactive analysis website. **D** Correlation analysis between ATF4 expression and ABCC1 expression in 48 PDAC tumor samples obtained in our institution. *R* = 0.3873, *p* = 0.0001. **E**, **F** qRT-PCR analysis revealed that silencing ATF4 expression inhibited ABCC1 expression but not ABCB1, ABCC2–5, or ABCG2 expression. **G** qRT-PCR analysis showed that TGF-β1 and CM-CAFs treatment upregulated ABCC1 expression, but SB431542 inhibited the promoting effect of CM-CAFs. **H** The luciferase assay indicated that the transcriptional activation of ABCC1 in PANC-1 cells was inhibited in ATF4-silenced cells and enhanced in TGF-β1- or CM-CAFs -treated cells with upregulated ATF4 expression. **I** Luciferase activity was detected in PANC-1 cells with mutations in the A1 site, A2 site, or both sites in the ABCC1 promoter region. Twenty-four hours after cotransfection with different human ABCC1 promoter-luciferase reporter constructs and pcDNA3.1-ATF4, all samples were harvested for luciferase activity experiments. The graph on the right side reveals the relative luciferase activity of the ABCC1 promoter. **J** ChIP assay was used to detect the directive binding of ATF4 to the ABCC1 promoter region. Specific antibodies against ATF4 were used for immunoprecipitation to harvest the protein-DNA complexes from PANC-1 cells. After cross-link reversal, the coimmunoprecipitated DNA was amplified by PCR using the indicated primers and resolved in 2% agarose gels. The graphs shown are representative results of three repeated assays and are presented as the mean ± SD. ^***^p < 0.001.

**Fig. 7:**
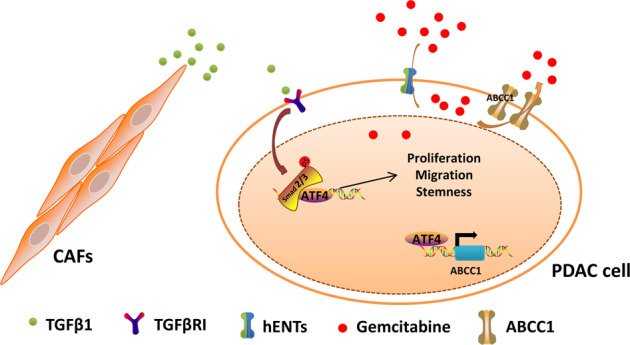
Diagram of the crosstalk between CAFs and pancreatic cancer cells. CAFs secrete TGF-β1 to facilitate SMAD2/3 phosphorylation followed by the upregulation of ATF4. Activation of ATF4 enhances proliferation, migration, and stemness in pancreatic cancer. Specifically, ATF4 directly binds to the ABCC1 promoter region and transactivates ABCC1, resulting in gemcitabine resistance.

Gemcitabine-based chemotherapy is an important part of PDAC treatment. We revealed that CAFs-enticed ATF4 participated in gemcitabine resistance in PDAC, but the mechanism is still unclear. Aberrant expression of the ATP-binding cassette (ABC) transport family is a vital mechanism causing chemotherapy resistance in multiple tumors. ATF4 induces drug resistance in acute myeloid leukemia (AML) by binding to the ABCB1 promoter^25^. To investigate the role of the ABC transport family in ATF4-centered gemcitabine resistance in pancreatic cancer, we analyzed the relationship between ATF4 mRNA expression and ABC transport family mRNA expression in the gene expression profiling interactive analysis website with TCGA and GTEx datasets. The positive correlation between ATF4 expression and ABCC1 expression (*R* = 0.37) was stronger than the correlations between ATF4 and ABCB1 (*R* = 0.23), ABCC2 (*R* = 0.22), ABCC3 (*R* = 0.34), ABCC4 (*R* = 0.29), ABCC5 (*R* = 0.24), and ABCG2 (*R* = 0.17). The correlation between CAF markers (ACAT2, FAP, PDGFRB) or TGF-β1 level and ABCC1 level was stronger than that with ABCB1 (Figs. 6A–C and S4). In the qRT-PCR assays of 48 PDAC tumor samples obtained in our department, a significant positive correlation was observed between ATF4 expression and ABCC1 expression (*R* = 0.3873, *p* = 0.0001, Fig. 6D). Silencing ATF4 expression downregulated ABCC1 levels in PANC-1 and BxPC-3 cells but not ABCB1, ABCC2, ABCC3, ABCC4, ABCC5, or ABCG2 (Fig. 6E, F). Moreover, TGF-β1 and CM-CAF treatment upregulated ABCC1 expression in PDAC cells, and SB431542 inhibited this effect (Fig. 6G). Using a luciferase activity assay, we revealed that the transcriptional activation of ABCC1 in PANC-1 cells was inhibited in ATF4-silenced cells and enhanced in TGF-β1- or CM-CAFs-treated cells with upregulated ATF4 expression (Fig. 6H). Two potential ATF4 binding sites (Fig. 6I, A1: −1420 bp to −1407 bp, ATATGGCGCAATGG; A2: −403 bp to −390 bp, ACAGGATGAAATGA) in the ABCC1 promoter region (−2000 bp to +1 bp) were predicted by using the JASPAR website^26^. To investigate the ATF4 binding site, site-directed mutagenesis in the A1 site and A2 site of the ABCC1 promoter was performed. Luciferase reporter assays demonstrated that mutating binding site A1 (−1420 bp to −1407 bp) did not affect ABCC1 promoter activity, while mutating the A2 site (−403 bp to −390 bp) or mutation of both sites abolished ABCC1 promoter activity (Fig. 6I), indicating that the 5′ upstream region (−403 bp to −390 bp) of the ABCC1 promoter was required for transcriptional activation by ATF4. Furthermore, a ChIP assay was performed to determine whether ATF4 directly binds to the ABCC1 promoter. The chromatin was precipitated with a specific ATF4 or IgG antibody and analyzed by PCR using primer sets that covered the entire length (−1700 bp to +53 bp) of the ABCC1 promoter. Consistent with the cotransfection studies, ChIP analysis showed that ATF4 binding was confined to the specific region of the ABCC1 promoter (−419 bp to−270 bp, Fig. 6J). In summary, these results demonstrate that ATF4 regulates ABCC1 expression through binding its specific promoter region and activating its transcription (Fig. 7).

## Discussion

CAFs are one of the most dynamic stromal cells in PDAC tissue and are characterized as accomplices and promoters of cancer malignancy and chemoresistance^27,28^. In the present work, we demonstrated that CAFs secrete TGF-β1 to upregulate the expression of ATF4 in pancreatic cancer cells via the SMAD2/3 pathway. ATF4 is overexpressed in PDAC and is associated with a poor prognosis. Downregulation of ATF4 expression inhibited proliferation, migration, sphere formation, and gemcitabine sensitivity in vitro. Subsequently, stably downregulating ATF4 expression suppressed tumor growth and enhanced gemcitabine sensitivity in vivo. Importantly, we demonstrated that ATF4 binds to the promoter of ABCC1 to activate ABCC1 transcription, which is a vital mechanism for the development of chemoresistance (Fig. 7).

ATF4, a stress response transcription factor, is involved in physiological homeostasis and cellular protection^10^. Recently, ATF4 was reported to be overexpressed in multiple tumors and associated with a poorer prognosis, including breast cancer^29^, osteosarcoma^30^, and esophageal squamous cell carcinoma^31^. Adrián et al. reported that ATF4 is overexpressed in triple-negative breast cancer and correlates with lower overall and relapse-free survival rates^11^. ATF4 depletion leads to the reduction of metastasis, cancer stemness, and tumor survival in vitro and in vivo and is involved in the TGFβ/SMAD and PI3K/mTOR pathways, playing growth factor-dependent functions. ATF4 is also reported to participate in regulatory networks of some biological behaviors in pancreatic cancer, including autophagy and hypoxia^12^. However, the specific role of ATF4 in the proliferation, migration, cancer stemness, and gemcitabine resistance of PDAC is still unclear. In our study, we found that ATF4 is overexpressed in PDAC tissues and pancreatic cancer cell lines. With cell proliferation assays, colony formation assays, Transwell assays, gemcitabine survival IC50 assays, and sphere formation assays, we demonstrated that silencing the expression of ATF4 could inhibit proliferation, migration, cancer stemness, and gemcitabine resistance. Clinical data showed that high ATF4 correlates with poor OS and PFS and serves as an independent prognostic factor. The lack of a downstream mechanism of ATF4 is one of the downsides in the present study.

The crosstalk between CAFs and ATF4 expression in cancer cells was then investigated. CAFs are very abundant in pancreatic tumor tissues. Due to the secretion of IL6, IL8, SDF-1, TGF-β1, and other cellular factors, CAFs contribute to the development of an inflammatory tumor environment^28^. ATF4 is one of the most important transcriptional mediators of the integrated stress response. Therefore, the CAF-mediated inflammatory response leads to the activation of ATF4. We demonstrated that TGF-β1 derived from CAFs upregulates the expression level of ATF4 in pancreatic cancer cells via the SMAD2/3 pathway. Further, we revealed that preventing crosstalk between CAFs and cancer cells partially inhibits cancer stemness and gemcitabine resistance and decreases the expression of metastasis-related genes such as MMP2, MMP9, and Snail.

Chemoresistance is a persistent challenge for pancreatic cancer clinical therapy. Gemcitabine-based single-agent or combinational chemotherapies remain the first-line therapies for most pancreatic cancer patients since the 1990s (refs. ^32,33^). Unfortunately, most patients experience treatment failure due to the development of chemoresistance. Wang et al. reported that ATF4-involved endoplasmic reticular stress blocks the apoptosis of PANC-1 cancer stem-like cells under gemcitabine treatment^34^. However, the mechanism by which ATF4 leads to gemcitabine resistance in PDAC remains unclear. Upregulation of ABCC1-5, ABCB1, and ABCG2 is the main reason for the development of multiple drug resistance in various cancers by excreting chemotherapy drugs out of cancer cells. ATF4 could bind to the ABCB1 promoter to activate transcription in AML (ref. ^25^). In our study, we first analyzed the correlation between ATF4 expression level and ABC transporter expression level. We found that the correlation between ATF4 level and ABCC1 level is stronger than that with ABCB1 level. Furthermore, the expression level of TGF-β1, ACAT2, FAP, or PDGFRB is positively associated with the expression level of ABCC1, while the correlations with ABCB1 are much weaker. In addition, ABCC1 expression is regulated by the level of ATF4 through silencing or CM-CAFs treatment. Mechanistically, we demonstrated the existence of an ATF4 binding motif in the ABCC1 promoter (−403 bp to −390 bp) through luciferase activity reporter assays and site-directed mutagenesis assays, and ChIP assays confirmed the directive binding of ATF4 to the ABCC1 promoter. Here, we showed direct evidence demonstrating that ATF4 mediates gemcitabine resistance by binding to the ABCC1 promoter for the first time.

In conclusion, we demonstrated that TGF-β1-positive CAFs are responsible for malignant progression and gemcitabine resistance, partially owing to paracrine induction of ATF4 via the SMAD2/3 pathway in PDAC cells. Our study provides the first evidence of the mechanism by which ATF4 contributes to gemcitabine resistance by transactivating ABCC1, suggesting an attractive therapeutic target for combating gemcitabine resistance in PDAC.

## Materials and methods

### Patients and clinical samples

PDAC tumor and paired normal pancreas samples were obtained from those who underwent surgery at Sun Yat-sen Memorial Hospital between January 2009 and January 2019. None received any neoadjuvant therapy before resection. Written informed consents have to be applicated for exemption, and the protocol was approved by the hospital’s Protection of Human Subjects Committee. The obtained samples were immediately snap-frozen in liquid nitrogen and transferred at −80 °C for further experiments. All samples were histologically confirmed with PDAC. The detailed clinicopathologic characteristics of patients are summarized in Table 1. The collection of follow-up data was carried out for all cases, and overall survival was defined as the time interval from the date of surgery to the date of death or the end-point of follow-up (May 2020).

### Cell culture

Human pancreatic cancer cell lines (CFPAC-1, AsPC-1, PANC-1, Capan-2, MIAPaCa-2, SW1990, and BxPC-3) and human pancreatic duct epithelial cell line (HPDE6C7) were purchased from the American Type Culture Collection (ATCC, Manassas, USA). Cells were cultured in Dulbecco’s modified Eagle’s medium (DMEM, Gibco, Thermo Fisher Scientific, CA, USA) containing 10% fetal bovine serum (FBS, Invitrogen, CA, USA) and 1% penicillin-streptomycin at 37 °C in humidified air with 5% CO_2_. As for co-culture with CAFs, PANC-1 and BxPC-3 cells were seeded in the lower chamber and CAFs were cultured in the upper chamber (0.4 μm, Corning) in 6-well plate. Two chambers were placed together the next day and then cultured for 3 days.

### Isolation of pancreatic CAFs and paired NFs

The isolation protocol was referred to in the previous description^22^. In brief, the fresh tissues were obtained from PDAC patients. The pancreatic tumor tissue and adjacent normal pancreas tissue (at least 2 cm from the outer tumor margin) were minced into 1–3 mm^3^ fragments, following digestion with 1 mg/ml collagenase I (CAT: C0130, Sigma) at 37 °C for 2 h. The solution and slag were centrifuged at 1000 rpm for 5 min and then washed with PBS (Gibco, Thermo Fisher Scientific, CA, USA) twice. The precipitation including isolated cells and cell masses were transferred and seeded in a 10-cm dish. Primary CAFs and NFs were cultured with DMEM plus 15% FBS and 1% penicillin-streptomycin and passaged for further experiments.

### RNA extraction and qRT-PCR

Total RNA from frozen pancreatic cancer tissues and corresponding non-neoplastic tissue or cultured cell lines were extracted with TRIzol reagent (Invitrogen, Carlsbad, USA) according to the manufacturer’s instructions. All primer sequences for the qRT-PCR assay were listed in Supplemental Table 1. For relative gene expression in tissues or cells, the levels were first normalized to GAPDH expression as ΔCt and then compared with corresponding control and converted to the fold changes (2^−ΔΔCt^).

### Half-maximal inhibitive concentration (IC50) assay

Cells were seeded in 96-well plates. The next day, fresh gemcitabine was added into cells at various concentrations of 0, 0.001, 0.01, 0.05, 0.1, 0.5, 1, 5, 10, 20, 50, 100, 500, and 1000 μM. After 72 h treatment, cells were photographed and quantified via CCK-8 assay. The half-maximal IC50 was calculated with software GraphPad Prism 7.

### Cell transfection and viral infection

The ATF4 siRNAs and synthetic sequence-scrambled siNC (GenePharma, Shanghai, China) were transiently transfected into pancreatic cancer cells using Lipofectamine 3000 (Invitrogen, USA). 24 h later, cells were collected for further experiments. The stable suppression of ATF4 was performed by short hairpin RNA interference. In total, 293T cells were used for virus packaging with a mixture of pHelper 1.0 vector (packaging plasmid) and pHelper 2.0 vector (envelope plasmid) (GeneChem, Shanghai, China) and Lipofectamine 2000 (Invitrogen) according to the manufacturer’s instructions. Lentiviruses were collected and transfected into pancreatic cancer cells. Stable cell lines were generated by treatment with puromycin (2 μg/ml) for at least 2 weeks. The sequences were listed in Supplemental Table 2.

### Cell proliferation and colony formation assay

For cell viability, pancreatic cancer cells (5000/well) were seeded in 96-well plates. After 1, 2, 3, and 4 days, 10 μl CCK-8 solution (Dojindo, Japan) was added and incubated at 37 °C for 2 h. Then, the absorbance was measured using a microplate reader (Tecan Trading AG, Switzerland) at 450 nm. Cell viability was calculated using GraphPad Prism 6.0 (La Jolla, USA) according to the manufacturer’s instructions. For colony formation assay, pancreatic cancer cells (1000/well) were cultured in 6-well plates for 2 weeks. Then, cell clusters were fixed with 4% paraformaldehyde for 15 min. After washed twice, cell clusters were stained with crystal violet for 20 min. After washed with moving water, cell clusters were counted.

### Transwell assay

Migration and invasion assays were performed in 24-well plates using Millicell culture plate inserts (Millipore, Darmstadt, Germany). For migration assay, 20,000 cells were seeded to the top chamber, and 10% FBS in DMEM was added to the bottom chamber. After 24 h, the cells that invaded through the membrane (migrated cells) were stained with crystal violet and counted in three fields under a 100× objective lens. For invasion assay, the top chambers were coated with Matrigel (1:8, Cat: 356234, BD, USA). After solidification, 40,000 cells were seeded in the top chamber, followed by the same procedure.

### Enzyme-linked immunosorbent (ELISA) assay

CAFs and NFs were cultured in DMEM medium without FBS for 2 days. Conditional mediums were collected, and secreted TGF-β1 was quantified using a Human TGF-β1 ELSIA Kit (E-EL-0162c, Elabscience, China) according to the manufacturer’s instructions.

### Sphere formation assay

PANC-1 and BxPC-3 cells were plated in 96-well ultra-low attachment plates (Corning, NY, USA) at a density of 1000 cells per well. Cells were maintained in serum-free DMEM/F-12 supplemented with 20 ng/ml human recombinant epidermal growth factor, 20 ng/ml basic fibroblast growth factor, and 1× B27 serum substitute; all from Invitrogen, Carlsbad, CA, USA. After incubated at 37 °C in 5% CO2 for 2 weeks, spheres with larger than 50 μm in diameter were counted.

### Immunofluorescence analysis

Cells were fixed with 4% paraformaldehyde and permeabilized in 0.1% Triton X-100. After incubation with primary antibody at 4 °C overnight, cells were incubated with secondary antibody in the dark, followed by cell nuclei staining with 4′,6-diamidino-2-phenylindole. Images were acquired using an LSM710 confocal microscope (Zeiss, Pleasanton, CA, USA). The primary antibodies are as following: α-SMA (Abcam, CAT: ab32575, 1:200), FAP (Abcam, CAT: ab28244, 1:200).

### Western blot analysis

Cells were lysed with RIPA (Beyotime, China) supplemental with a protease inhibitor cocktail (Roche). Protein lysate (30 μg) was separated by SDS-PAGE and transferred to a PVDF membrane (Bio-Rad, USA). After blocking, immunodetection was performed using primary and HRP-linked secondary antibodies, followed by detection with an enhanced ECL system (Pierce). The primary antibodies are as following: ATF4 (Proteintech, CAT: 10835-1-AP, 1:1000), ABCC1 (Proteintech, CAT:67228-1-Ig, 1:10,000), ABCB1 (Proteintech, CAT: 22336-1-AP, 1:500), GAPDH (Proteintech, CAT: 10494-1-AP, 1:20,000), β-actin (Proteintech, CAT: 60008-1-Ig, 1:5000) and p-SMAD2/3 (Immunoway, CAT: YP0362, 1:1000).

### Immunohistochemistry

Histologic sections from formalin-fixed, paraffin-embedded tissues were subjected to antigen retrieval in citrate buffer for 15 min, followed by blocking in normal goat serum for 30 min. Then tissue sections were incubated with primary antibody (ATF4: Proteintech, CAT: 10835-1-AP, 1:200) at 4 °C overnight. Avidin–biotin peroxidase detection systems with DAB substrate were used to mark the locations of antigens, followed by counterstaining with hematoxylin. Immunohistochemical signal intensity and positively stained field of tissue sections were evaluated and scored independently by two observers.

### Luciferase reporter assay and site-directed mutagenesis

The ABCC1 promoter (−1700 bp to +53 bp in pGL2-basic) is purchased from GenePharma Bio Co. Ltd. (Shanghai, China). The resulting construct was confirmed by DNA sequencing. Reporter gene assays were performed as the manufacturer described. The pGL2-Basic plasmid containing human wild type ABCC1 promoter sequence (−1700 bp to +53 bp) was used as a template, and putative binding site mutagenesis kits were performed with the GenePharma Bio Co. Ltd. (Shanghai, China). Two potential ATF4 binding sites (A1: −1420 bp to −1407 bp; A2: −403 bp to −390 bp) in the ABCC1 promoter region (−2000 bp to +1 bp) were predicted on the JASPAR website. The wild type ABCC1 promoter sequence was mutated as following: MUT1: 5′–ATATGGCGCAATGG–3′ (−1420/−1407 bp) was mutated to 5′–GCGCAATATGGCAA–3′; MUT2: 5′–ACAGGATGAAATGA-3′ (−430/−390 bp) was mutated to 5′–GTGAAGCAGGGCAG–3′.

### Chromatin immunoprecipitation (ChIP) assay

ChIP assays were performed using EZ-Magna ChIP Chromatin Immunoprecipitation Kit according to the manufacturer’s instructions. Briefly, 5 × 10^6^ indicated PANC-1 cells were fixed in 1% formaldehyde for 10 min at room temperature. After fixed, cells were collected, lysed, and sonicated for ten cycles with a Sonics VCX130. Antibodies against ATF4 (5 µl per 1 mg total protein; Proteintech, CAT: 10835-1-AP, 1:200) and rabbit IgG (2 µl per 1 mg total protein) were used for immunoprecipitation. Analysis of the PCR products was performed on the precipitated DNA from a standard 2% (w/v) agarose gel by electrophoresis in Tris-acetate EDTA buffers. The primer sequences used in the ChIP assay are listed in Table S3.

### Animal study

Six-week-old male Balb/c nude mice were purchased and maintained at the Laboratory Animal Center of Sun Yat-sen University in a specific pathogen-free environment. The animal care and experimental protocols were approved by the Institutional Guidelines of Guangdong Province and by the Use Committee for Animal Care. PANC-1 cells (2 × 10^6^) with stably ATF4 knockdown or mock cells were subcutaneously transplanted into the bilateral hind legs of mice. Tumor volumes were assessed every 5 days, using a standard formula (volume = length × width^2^/2). For gemcitabine treatment analysis, all mice received an intraperitoneal injection with gemcitabine (100 mg/kg, twice a week) one week after transplant. Thirty days later, mice were sacrificed, tumors were harvested and immediately snap-frozen in liquid nitrogen or fixed with formalin for further analysis.

### Graphs and statistical analysis

All graphs were made using GraphPad Prism 7.0 (GraphPad Software Inc, CA). All data are expressed as means ± standard deviations of at least three independent experiments. One-way analysis of variance (ANOVA) or Student’s *t*-test was performed to compare groups. Survival was measured according to the Kaplan–Meier method and analyzed by the log-rank test. The associations between clinicopathological characteristics or α-SMA expression and ATF4 expression were analyzed using the χ2 test. Differences were considered significant for a *p*-value of *p < 0.05, **p < 0.01, ***p < 0.001, respectively.

## Supplementary information

Figure S1

Figure S2

Figure S3

Figure S4

supplemental figure legend

Table S1

Table S2

Table S3
