# Supplementary figures and images for: Cancer-associated fibroblasts-mediated ATF4 expression promotes malignancy and gemcitabine resistance in pancreatic cancer via the TGF-β1/SMAD2/3 pathway and ABCC1 transactivation
Source: Cell Death Dis. 2021 Mar 29;12(4):334. doi: 10.1038/s41419-021-03574-2 (PMC8007632; doi:10.1038/s41419-021-03574-2)

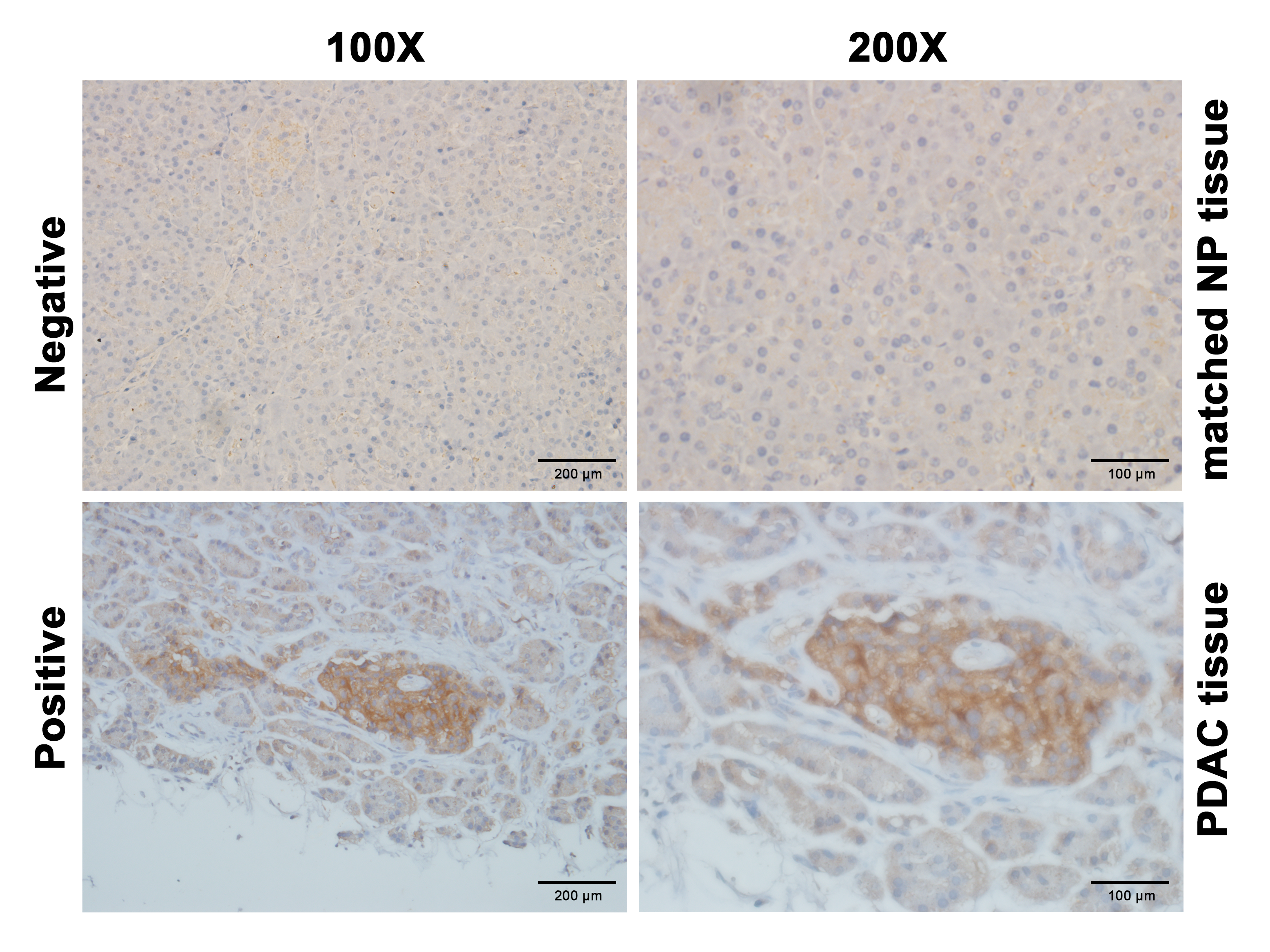

Supplement: Supplementary file 1 — Figure S1 [file 41419_2021_3574_MOESM1_ESM.tif]

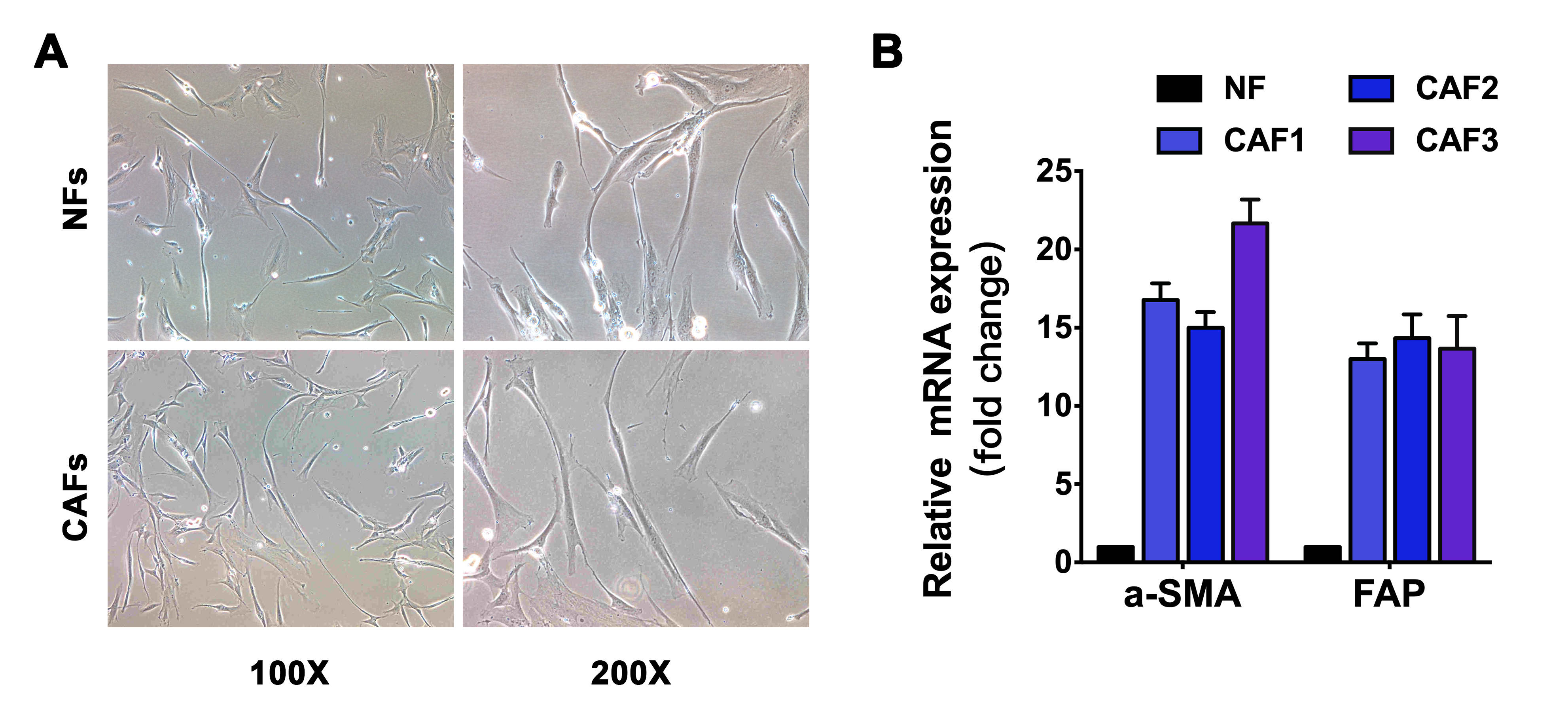

Supplement: Supplementary file 2 — Figure S2 [file 41419_2021_3574_MOESM2_ESM.tif]

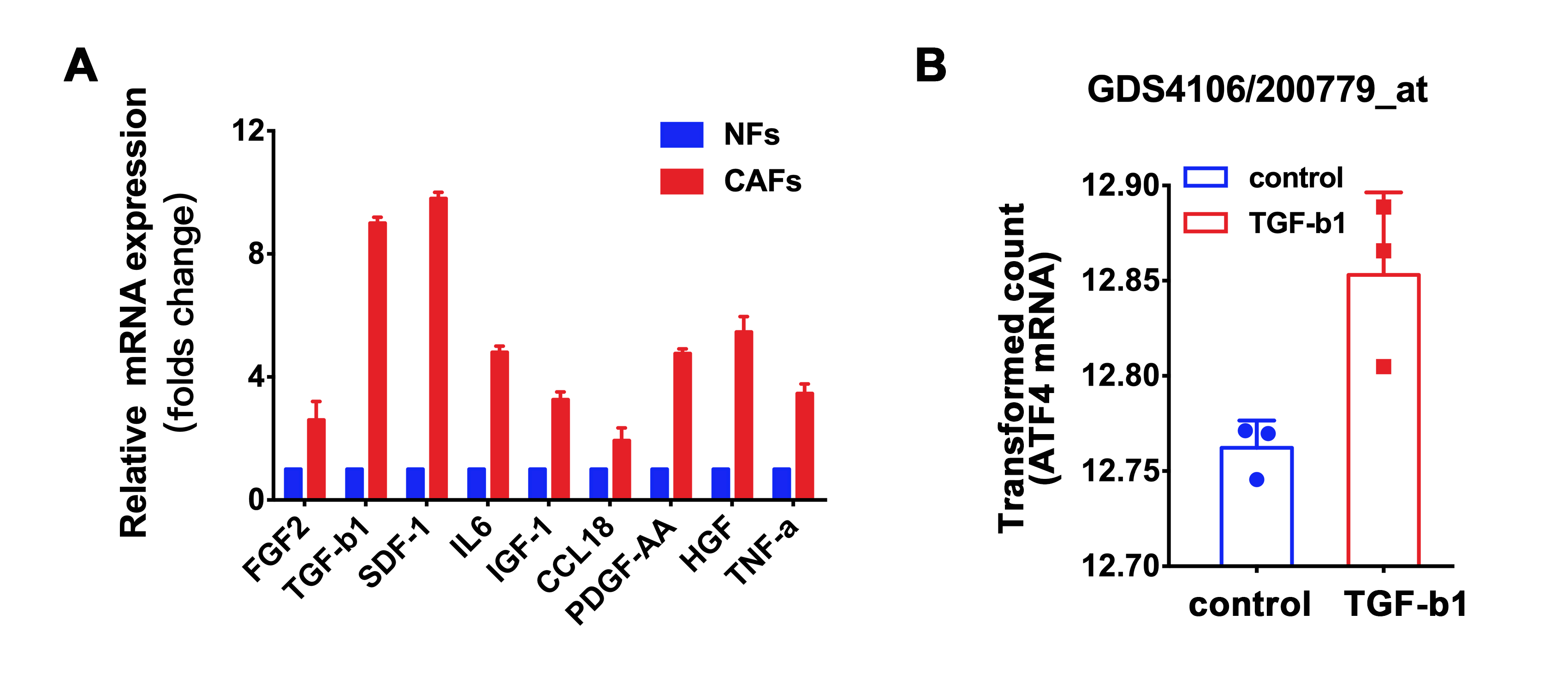

Supplement: Supplementary file 3 — Figure S3 [file 41419_2021_3574_MOESM3_ESM.tif]

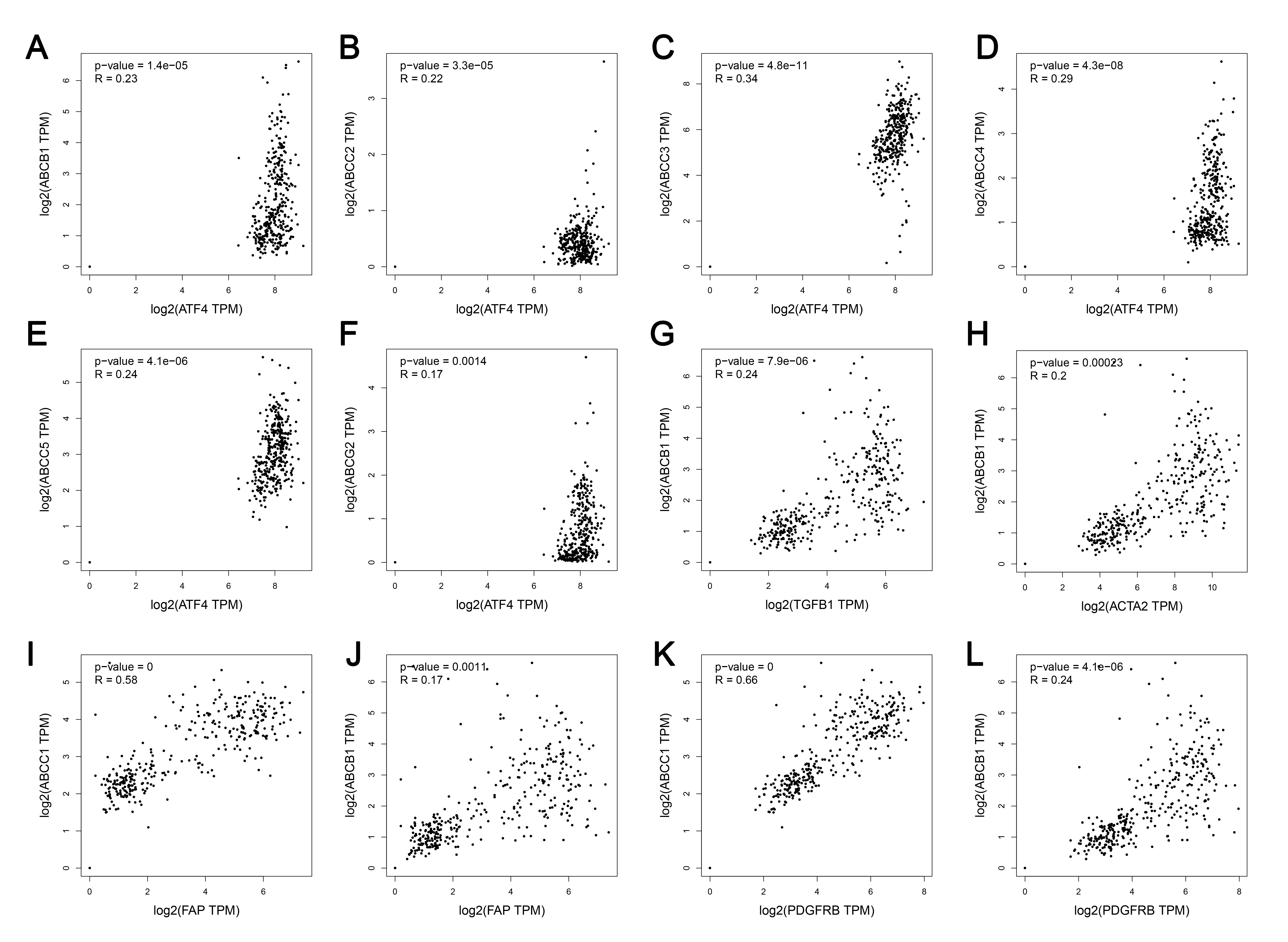

Supplement: Supplementary file 4 — Figure S4 [file 41419_2021_3574_MOESM4_ESM.tif]
