# Supplementary material for: Cancer-associated fibroblasts-mediated ATF4 expression promotes malignancy and gemcitabine resistance in pancreatic cancer via the TGF-β1/SMAD2/3 pathway and ABCC1 transactivation
Source: Cell Death Dis. 2021 Mar 29;12(4):334. doi: 10.1038/s41419-021-03574-2 (PMC8007632; doi:10.1038/s41419-021-03574-2)
Supplement: Supplementary file 5 — supplemental figure legend [file 41419_2021_3574_MOESM5_ESM.docx]

**Supplemental figure legends:**

**Figure S1.** Representative IHC staining images showed ATF4 expression in PDAC tissue and matched normal pancreas tissue.

**Figure S2.** **A.** Morphological images of NFs and CAFs isolated from fresh pancreatic normal tissue and tumor samples from PDAC patients. **B.** qRT-PCR analysis of the expression of a-SMA and FAP in NFs and CAFs. CAF1-3 were isolated from 3 PDAC patients.

**Figure S3. A.** qRT-PCR assay showed the expression of FGF2, TGF-β1, SDF-1, IL-6, IGF-1, CCL18, PDGF-AA, HGF and TNF-α in NFs and CAFs. **B.** The expression profile of GDS4106/200779-at.

**Figure S4.** Correlation analysis between the expression of two genes was performed using the GEPIA website.
