# Supplementary material for: Cancer-associated fibroblasts-mediated ATF4 expression promotes malignancy and gemcitabine resistance in pancreatic cancer via the TGF-β1/SMAD2/3 pathway and ABCC1 transactivation
Source: Cell Death Dis. 2021 Mar 29;12(4):334. doi: 10.1038/s41419-021-03574-2 (PMC8007632; doi:10.1038/s41419-021-03574-2)
Supplement: Supplementary file 6 — Table S1 [file 41419_2021_3574_MOESM6_ESM.docx]

**Supplemental table 1. The primer sequences for qRT-PCR.**

| **Gene** | **Sense (5’ to 3’)** | **Anti-sense (5’ to 3’)** |
| --- | --- | --- |
| ATF4 | ATGACCGAAATGAGCTTCCTG | GCTGGAGAACCCATGAGGT |
| GAPDH | GGAGCGAGATCCCTCCAAAAT | GGCTGTTGTCATACTTCTCATGG |
| ABCC1 | CTCTATCTCTCCCGACATGACC | AGCAGACGATCCACAGCAAAA |
| ABCB1 | TTGCTGCTTACATTCAGGTTTCA | AGCCTATCTCCTGTCGCATTA |
| a-SMA | AAAAGACAGCTACGTGGGTGA | GCCATGTTCTATCGGGTACTTC |
| FAP | ATGAGCTTCCTCGTCCAATTCA | AGACCACCAGAGAGCATATTTTG |
| ALDH1A1 | GCACGCCAGACTTACCTGTC | CCTCCTCAGTTGCAGGATTAAAG |
| MMP2 | TACAGGATCATTGGCTACACACC | GGTCACATCGCTCCAGACT |
| MMP9 | TGTACCGCTATGGTTACACTCG | GGCAGGGACAGTTGCTTCT |
| Snail | TTCCAGCAGCCCTACGACCAG | GCCTTTCCCACTGTCCTCATC |
| ABCC2 | CCCTGCTGTTCGATATACCAATC | TCGAGAGAATCCAGAATAGGGAC |
| ABCC3 | TGGGGTGAAGTTTCGTACTGG | CACGTTTGACTGAGTTGGTGATA |
| ABCC4 | AGCTGAGAATGACGCACAGAA | ATATGGGCTGGATTACTTTGGC |
| ABCC5 | AGTCCTGGGTATAGAAGTGTGAG | ATTCCAACGGTCGAGTTCTCC |
| ABCG2 | CAGGTGGAGGCAAATCTTCGT | ACCCTGTTAATCCGTTCGTTTT |
| TGFb1 | GGCCAGATCCTGTCCAAGC | GTGGGTTTCCACCATTAGCAC |
| FGF2 | GCGAATTCATGGCCGCCGGGAGCATCAC | CGCTCGAGTCAGCTCTTAGCAGACATTG |
| SDF-1 | CCCGAAGCTAAAGTGGATTC | TTCAGAGCTGGGCTCCTACT |
| IL-6 | ATGAACTCCTTCTCCACAAGC | CTACATTTGCCGAAGAGCCCTCAGGCTGGACTG |
| IGF-1 | CTAGGCACTCTGCTTGC | CTTGGGCATGTCAGTGTGGC |
| CCL18 | AAACTCGAGCTGCCCAGCATCATGAAGG | TTTGGATCCCCTCAGGCATTCAGCTTCAG |
| PDGF-AA | GATACCTCGCCCATGTTCTG | TGGCACTTGACTGCTCGT |
| HGF | TACTGCAGACCAATGTGCTA | GAATTTGTGCCGGTGTGGTG |
| TNF-a | ATGAGCACTGAAAGCATGATC | TCACAGGGCAATGATCCCAAAGTAGACCTGCCC |
