# Supplementary material for: Cancer-associated fibroblasts-mediated ATF4 expression promotes malignancy and gemcitabine resistance in pancreatic cancer via the TGF-β1/SMAD2/3 pathway and ABCC1 transactivation
Source: Cell Death Dis. 2021 Mar 29;12(4):334. doi: 10.1038/s41419-021-03574-2 (PMC8007632; doi:10.1038/s41419-021-03574-2)
Supplement: Supplementary file 7 — Table S2 [file 41419_2021_3574_MOESM7_ESM.docx]

**Supplemental table 2. The Oligonucleotide sequences.**

| **Gene** | **sense (5’ to 3’)** | **Anti-sense (5’ to 3’)** |
| --- | --- | --- |
| siNC | UUCUCCGAACGUGUCACGUTT | ACGUGACACGUUCGGAGAATT |
| siATF4-1 | GGGUAUAGAUGACCUGGAATT | UUCCAGGUCAUCUAUACCCTT |
| siATF4-2 | GGUGAACCCAAUUGGCCAUTT | AUGGCCAAUUGGGUUCACCTT |
