# Supplementary material for: Cancer-associated fibroblasts-mediated ATF4 expression promotes malignancy and gemcitabine resistance in pancreatic cancer via the TGF-β1/SMAD2/3 pathway and ABCC1 transactivation
Source: Cell Death Dis. 2021 Mar 29;12(4):334. doi: 10.1038/s41419-021-03574-2 (PMC8007632; doi:10.1038/s41419-021-03574-2)
Supplement: Supplementary file 8 — Table S3 [file 41419_2021_3574_MOESM8_ESM.docx]

**Supplemental table 3. The primer sequences used in the ChIP assay**

| **Amplicon** | **Sense (5’ to 3’)** | **Anti-sense (5’ to 3’)** |
| --- | --- | --- |
| -1700/-1460 | ATCCTATCTACCTTCCTCCTT | CCTCTCCTCCTTAATCTCCT |
| -1500/-1250 | TCCTCTGTCTCTACCTGTC | CTTTGAACCCAAGAAGTCTG |
| -1277/-1044 | AAGAACACAGCCTTAGGAA | AACAGGAGAATCACTTGAAC |
| -1050/-800 | TCTTGAACTCCTGACCTTATG | ACTGAAGCCTGGAGACAT |
| -816/-603 | CACAGGTGAGGTCAACAG | GGTTCAGAAAGGCGAAGT |
| -614/-410 | AGTCACTTCGCCTTTCTG | GTCACCTGGCTAATCACTT |
| -419/-270 | GGAGACTTTACAGGATGAAATG | ACAGTATCCGTCACCAGG |
| -293/+53 | AAAGTGGTCGCAGGGTGTG | CCAGATCCTCCAAGGCTTAG |
